# Comparison of two RT-qPCR methods targeting BK polyomavirus microRNAs in kidney transplant recipients

**DOI:** 10.3389/fmed.2023.1281625

**Published:** 2023-11-28

**Authors:** Kenza Zoubir, Véronique Descamps, Aurélien Aubry, Francois Helle, Catherine Francois, Sandrine Castelain, Etienne Brochot, Baptiste Demey

**Affiliations:** ^1^Laboratoire de Virologie, Centre Hospitalier Universitaire Amiens-Picardie, Amiens, France; ^2^UR UPJV 4294, Agents Infectieux, Résistance et Chimiothérapie (AGIR), Centre Universitaire de Recherche en Santé, Université de Picardie Jules Verne, Amiens, France

**Keywords:** BK polyomavirus, microRNA, RT-qPCR, kidney transplantation, biomarker, comparison

## Abstract

**Background:**

BK polyomavirus replication leads to progressive tubulointerstitial nephritis and ureteral stenosis, with a considerable risk of subsequent graft failure in kidney transplant recipients. Since specific antiviral therapies are lacking, new tools are required to enhance the biological monitoring of the infection. Viral microRNAs are promising new biomarkers, but the performance of RT-qPCR methods limits the clinical application and the validation of a standard method for quantification.

**Methods:**

We compared *TaqMan microRNA Assays* and *TaqMan Advanced miRNA Assays* for bkv-miR-B1-3p and bkv-miR-B1-5p quantification in synthetic microRNA templates and in 44 urine samples belonging to 14 consecutive kidney transplant recipients with BK polyomavirus replication from Amiens University Medical Center in a 1-year span.

**Results:**

Cycle threshold values were constantly higher with *TaqMan Advanced MicroRNA Assays*. *TaqMan microRNA Assays* showed better performance in predicting the good prognosis of BK polyomavirus nephropathy.

**Conclusion:**

Overall, *TaqMan MicroRNA Assays* appeared to be a more sensitive and accurate RT-qPCR method than *TaqMan Advanced MicroRNA Assays* to quantify bkv-miR-B1-3p and bkv-miR-B1-5p BKPyV miRNAs in patients’ urine samples.

## 1 Introduction

The BK polyomavirus (BKPyV) is a ubiquitous human *Polyomaviridae* in the general population (1). Specific antibodies against this virus can be found in at least 70–80% of adults’ sera (2, 3). Primary infection usually occurs in childhood with light to mild symptoms. After the viremic phase, virions persist in various tissues, especially the kidney and bladder. In immunocompetent individuals, the life-long viral replication remains inconspicuous, without clinical manifestations, and urinary viral shedding can periodically be detected in asymptomatic subjects (4). In case of immunosuppression, BKPyV replication can exacerbate and lead to severe outcomes. Patients who suffer the most from intense BKPyV replication are kidney transplant recipients (KTRs) (5). Under the influence of individual characteristics, the type of immunosuppressive therapy, and transplantation procedures, the detection of BKPyV is significantly higher in KTRs than in the general population (6). In fact, the presence of BKPyV DNA in urine (DNAuria) is found in half of these patients (5, 7). Sustained and high-level DNAuria reflects the intensity of viral replication and kidney tissue damage, also marked by the passage of viral DNA in the systemic bloodstream (DNAemia). Sustained BKPyV DNAemia and/or DNAuria with high levels (>10^4^ and 10^7^ copies/mL, respectively) are presumptive for BKPyV nephropathy (BKVN) and irreversible allograft lesions (8–10). However, proven BKVN can only be assessed by immunostaining of viral proteins on a kidney transplant biopsy (11–13). Such an invasive diagnostic method is not constantly applicable. Moreover, the frequency and quality of biopsy puncture are variable between centers and physicians, which makes viral load monitoring a more reliable diagnostic strategy. BKVN is a clinical presentation of the BKPyV infection that concerns 5–7% of the KTRs and leads to graft loss in most cases (10). To date, there is no prophylactic or curative treatment against BKVN, so the only therapeutic intervention to manage the infection is the lightening of immunosuppressants (14, 15). The time point for therapy adjustment is debated and not consensual; this may start with the onset of high-level DNAuria, DNAemia, high-level DNAemia, or clinico-biological signs of renal tissue damage. However, prolonged insufficient immunosuppression after kidney allograft increases the risk of immunological rejection and limits the medical possibilities to regulate BKPyV infection (16). Moreover, the conditions for initial dose restoration are not clearly defined, and robust markers are still missing to assess the control of the infection (10, 17, 18). The latest recommendations suggest monitoring the BKPyV DNAemia level, but it could remain detectable for months after BKVN resolution, underlying the need for advanced viral marker biosensing methods (10). Recent studies described the potential of viral micro-RNA (miRNA) quantification in urine as a new non-invasive biomarker to monitor BKPyV infection and predict the risk of BKVN (19–25).

BKPyV is a virus with circular double-stranded DNA that replicates in two directions from a unique origin, inserted in the non-coding control region (26). The viral genome sequentially encodes early proteins (the large T, small T, and truncated T antigens) that initiate viral replication, and then the late proteins implicated in the structural assembly of virions (VP1, VP2, VP3, and Agno proteins) (27). Transcription in the direction of late genes is also responsible for the generation of a pre-miRNA whose sequence is perfectly complementary to the large T antigen sequence (28). Recently, it was shown that RNA polymerase II could circle the genome multiple times, producing transcripts with tandem repeats of BKPyV sequences (29). This phenomenon places the pre-miRNA sequence in a genome-sized intron and promotes a higher miRNA expression level. A typical mechanism of maturation involving Dicer and Drosha activity leads to the generation of two mature miRNAs: bkv-miR-B1-3p and bkv-miR-B1-5p (30). The bkv-miR-B1-3p sequence is identical to the JC polyomavirus (JCPyV) 3p-miRNA, but the bkv-miR-B1-5p is specific to the BKPyV, with a highly conserved sequence (31). The functions of these molecules are not fully understood, but they are suspected to play a critical role in viral persistence by the autoregulation of viral replication and modulation of the immunological host response (32, 33).

MiRNAs, including those from BKPyV, are highly stable in biological fluids such as urine (34). These 22 nucleotide-long molecules are protected from RNAses by cargo proteins such as Ago2 in the extracellular environment, facilitating their detection in laboratory medicine (35–37). It has been shown that bkv-miR-B1-3p and bkv-miR-B1-5p are expressed earlier than viral DNA replication after the initiation of BKPyV replication in cells (32, 38). Moreover, the extracellular level of bkv-miR-B1-5p was shown *in vitro* to be linked to the active replication and infectivity of BKPyV (34). On the contrary, viral DNA is the standard biomarker to monitor the infection, but highly detectable BKPyV DNA in the urine and plasma can turn out to be a defective viral genome, unspecific to active viral replication (39). In some cases, a high BKPyV load may not reflect active replication and might accidentally mislead therapeutic adjustment. Thus, a study previously suggested that BKPyV miRNA could be a more specific biomarker of viral replication and infectivity than DNA during chronic infection (23). The authors concluded that these viral transcripts could constitute a promising new tool for viral monitoring and drug management in a personalized medicine strategy.

Several procedures for BKPyV miRNA quantification in KTRs have been described, depending on the purpose of the studies, but authors rarely compare results from distinct methods. To date, no consensus method exists for the clinical application of viral miRNA quantification. Hence, evaluation and comparison of various methods, notably in *ex vivo* samples, are required to reach a standardized method. The present article describes the intrinsic performance of two reverse-transcription quantitative polymerase chain reaction (RT-qPCR) kits, *TaqMan microRNA Assays* and *TaqMan Advanced miRNA Assays* (both obtained from Applied Biosystems, Pleasanton, CA, USA), for synthetic bkv-miR-B1-3p and bkv-miR-B1-5p quantitative measurements. Results of longitudinal viral miRNA quantification in the 44 urine samples from 14 KTRs diagnosed for probable BKVN were also compared between the methods.

## 2 Patients and methods

### 2.1 Study design and population

The study comprised 14 patients who had undergone kidney transplantation between 5 July 2017 and 5 July 2018, at the Amiens University Medical Center (Amiens, France). The whole patient population developed a probable BKVN during the first year of follow-up, marked by high-level BKPyV DNAuria and BKPyV DNAemia, as defined by the American Society of Transplantation Infectious Diseases Community of Practice (10). The other main inclusion criteria were age 18 or older and regular follow-up at the medical center during the first post-transplantation year. Patients who experienced graft failure (transplant removal or requirement for dialysis) or died during the follow-up period were excluded. The present retrospective study was based on prospectively collected, de-identified biological samples, and clinical information for the 14 selected KTRs. A total of 127 urine samples and 145 plasma samples were analyzed. BKPyV DNA loads were prospectively measured during the usual follow-up of KTRs, and the data were obtained from the patients’ clinical records. Among the 127 urine samples, 44 samples positive for BKPyV DNA were retrospectively analyzed for BKPyV miRNA, which was the purpose of the study. The investigators who assayed the pairs of concomitantly collected urine and plasma samples for BKPyV miRNAs were blinded to the patients’ medical history and the BKPyV DNAemia results. The samples used for the present study were leftover urine samples collected during routine patient management. As bkv-miR-B1-3p is strictly identical to the 3p-miRNA of JCPyV, the urine samples were checked for the absence of JCPyV DNA using PCR before inclusion, as previously described (40).

The study was conducted according to the guidelines of the Declaration of Helsinki and approved by the Ethics Committee of the Amiens University Medical Center (protocol code PI2022_843_0078, 12 May 2022). Informed consent was obtained from all subjects involved in the study.

### 2.2 Nucleic acid extraction

Viral DNA was extracted, as previously described (23), from 200 μL of urine or plasma. A measure of 5 μL of a specific internal inhibition control (IC) was added to the lysis buffer and simultaneously purified with viral DNA using specific protocol B in the NucliSENS easyMAG system (bioMérieux, Marcy-l’Etoile, France). The extracted DNA was eluted with 50 μL of elution buffer and frozen at −80°C before use.

miRNAs were extracted from 400 μL of urine with the miRNeasy Serum/Plasma Advanced Kit (Qiagen, Venlo, The Netherlands). cel-miR-39 (4iRbase accession number: MI0000010) was used as an IC. Synthetic cel-miR-39 corresponds to the miRNeasy Serum/Plasma Spike-In Control (Qiagen; reference: 219610). A total of 5.6 × 10^8^ copies of synthetic cel-miR-39 were added to each sample, as recommended by the manufacturer. The miRNA-enriched total RNA was extracted with a QIAcube device (Qiagen), according to the manufacturer’s protocol. The miRNAs were eluted with 30 μL of RNAse-free water, and the solution was frozen at −80°C before use.

### 2.3 Real-time PCR assay for BKPyV DNA

BKPyV DNA was amplified and quantified with a RealStar BKV PCR Kit (Altona Diagnostics, Hamburg, Germany), as described previously (41).

### 2.4 miRNA RT-PCR assays

*TaqMan microRNA Assays* were performed as previously described (23): Reverse transcription (RT): *TaqMan® MicroRNA Reverse Transcription Kit* (Applied Biosystems) was used to specifically reverse-transcribe BKPyV miRNAs and an IC, according to the manufacturer’s instructions. RT primers for miRNAs were provided with specific *TaqMan MicroRNA Assays* (Applied Biosystems): 006801_mat for bkv-miR-B1-3p, 007796_mat for bkv-miR-B1-5p, and 000200 for cel-miR-39. For each miRNA and each sample, RT was performed with 5 μL of miRNA-enriched eluate and 10 μL of RT mix (prepared as recommended by the manufacturer). RT reactions were performed with a Veriti (Applied Biosystems) thermocycler in accordance with the kit’s instructions. Complementary DNA specimens were frozen at −20°C before use. Real-time PCR: the reaction mix contained 1 μL of TaqMan MicroRNA Assay probes (Applied Biosystems), 10 μL of TaqMan Universal PCR Master Mix (Applied Biosystems), 7 μL of RNAse-free water, and 2 μL of cDNA specimen. Real-time PCR mix was prepared according to the manufacturer’s instructions, and PCR reaction cycles were performed in 96-well plates with the QuantStudio 5 System (Applied Biosystems). Signals were measured and analyzed using QuantStudio software (Applied Biosystems). For the sake of readability, this assay will be called a *“classic” assay* for the remainder of this article.

*TaqMan Advanced miRNA Assays*: Reverse transcription (RT): *TaqMan Advanced miRNA cDNA Synthesis Kit* (Applied Biosystems) was used to randomly reverse-transcribe the whole miRNAs contained in eluate, including BKPyV miRNAs and cel-miR-39, according to the manufacturer’s instructions. For each miRNA and each sample, the first RT protocol step was performed with 2 μL of miRNA-enriched eluate. RT reactions were performed with a Veriti (Applied Biosystems) thermocycler in accordance with the kit’s instructions. Complementary DNA was pre-amplified as recommended by the manufacturer, and specimens were frozen at −20°C before use. Real-time *FAST Advanced* PCR mix (Applied Biosystems) was prepared according to the manufacturer’s instructions, and PCR reaction cycles were performed in 96-well plates with the QuantStudio 5 System (Applied Biosystems). Signals were measured and analyzed using QuantStudio software (Applied Biosystems). For the sake of readability, this assay will be called *“advanced” assay* for the remainder of this article.

The quality of miRNA purification was assessed by the qualitative detection of the IC (cel-miR-39 spike-in control obtained from Qiagen) using the two RT-PCR methods.

Quantities in eluate were calculated from the cycle threshold (Ct) results relative to standards diluted in the concentration range of 10^1^ to 10^7^ molecules/microliter. The standards were the synthetic oligonucleotide sequences of bkv-miR-B1-3p and bkv-miR-B1-5p (UGCUUGAUCCAUGUCCAGAGUC and AUCUGAGACUUGGGAAGAGCAU; catalog numbers: MSY0009150 and MSY0009149; Qiagen). The results presented in the tables and figures were expressed as the number of copies (or log_10_ copies) per mL of urine, based on the eluate/initial urine volume ratio.

### 2.5 Definitions

Patients with probable BKVN were categorized on the basis of the infection progression and published nomenclature (10).

A resolved BKVN case was defined as “undetectable BKPyV DNAemia” or “decreasing BKPyV DNAemia (> −1 log_10_ copies/mL from diagnosis) associated with low-level BKPyV DNAuria (<7 log_10_ copies/mL)” at the end of the 1-year post-transplantation follow-up.

A sustained BKVN case was defined as “stable or raising of BKPyV DNAemia and BKPyV DNAuria levels” during the period of follow-up.

### 2.6 Statistical analyses

Graphs and statistical analyses were performed using GraphPad Prism version 8.0.0 for Windows (GraphPad Software, San Diego, CA, USA). Correlations between parameters were studied with Pearson’s test. Fisher’s exact test was performed to compare the proportions of populations. The threshold for statistical significance was set to a value of *p* < 0.05.

## 3 Results

### 3.1 Intrinsic performance of RT-qPCR assays

**Figure 1:**
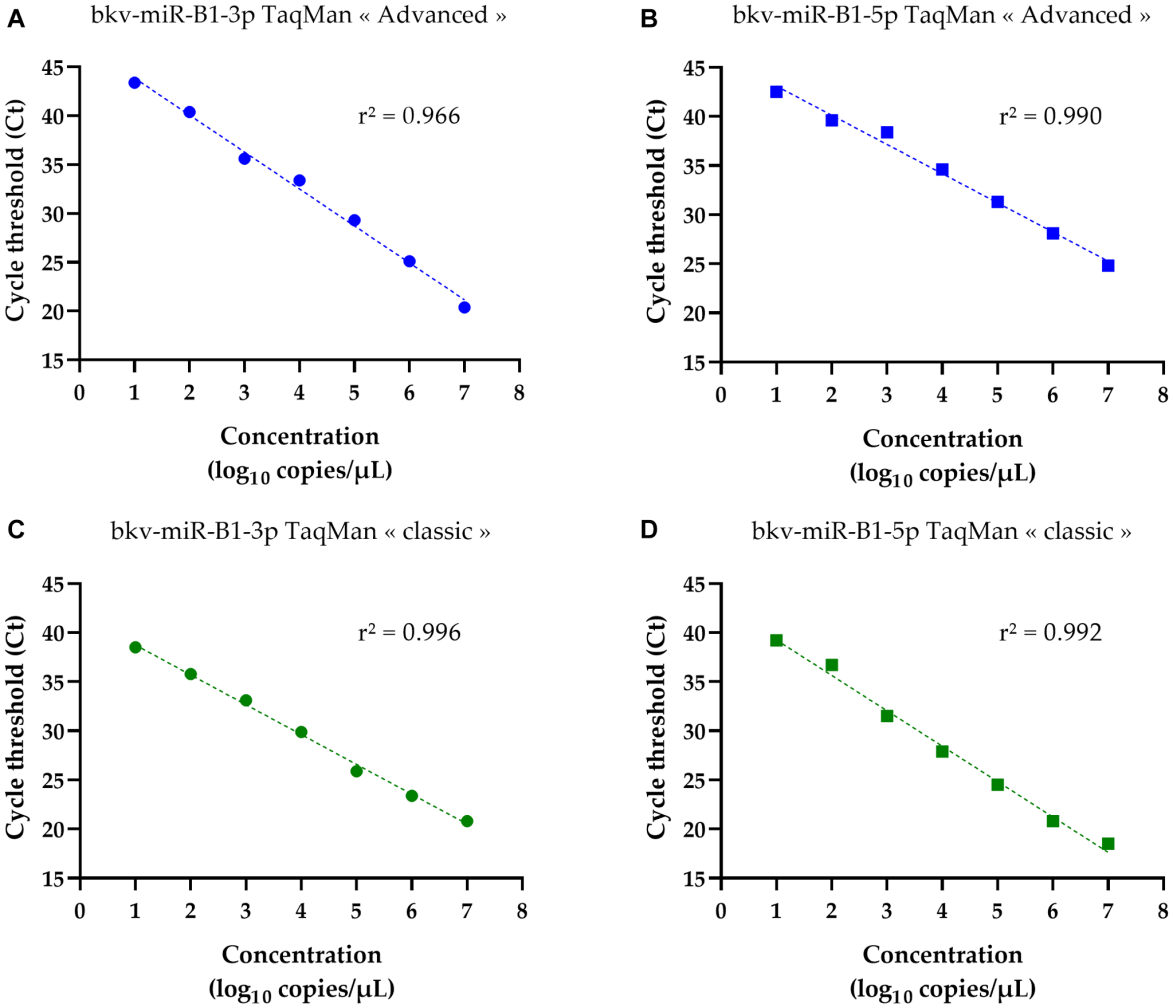
Standard curves of bkv-miR-B1-3p and bkv-miR-B1-5p RT-PCR assays. For each miRNA, RT-PCR was performed with *TaqMan Advanced MicroRNA Assays*® (called “advanced”) and *TaqMan MicroRNA Assays*® (called “classic”). Curves **(A)** and **(B)** correspond to quantification of bkv-miR-B1-3p and bkv-miR-B1-5p standards, respectively, with *TaqMan Advanced MicroRNA Assays*®. Curves **(C)** and **(D)** correspond to quantification of bkv-miR-B1-3p and bkv-miR-B1-5p standards, respectively, with *TaqMan MicroRNA Assays*®.

In a previous study, the authors discussed the limits of quantification of *TaqMan microRNA assay* that could have underestimated the proportions of samples being positive for BKPyV miRNA (23). The above-introduced *TaqMan Advanced miRNA Assays* may be a solution to enhance the sensitivity of BKPyV miRNA detection. To validate the efficiency of miRNA RT-PCR assays, the synthetic oligonucleotide sequences of bkv-miR-B1-3p and bkv-miR-B1-5p were used as templates. Serial dilutions from 7 log_10_ copies/μL to 1 log_10_ copies/μL were prepared for both miRNAs in RNAse-free water. The Ct obtained by RT-PCR analysis was correlated with miRNA concentrations for bkv-miR-B1-3p and bkv-miR-B1-5p, regardless of the assay (Figure 1). For bkv-miR-B1-3p quantification, the linear correlation coefficient *r*^2^ was equal to 0.966 with TaqMan “advanced” assay. A better *r*^2^ (0.996) was obtained for bkv-miR-B1-3p quantification with TaqMan “classic” assay. Linear correlation coefficients r^2^ were comparable between “advanced” and “classic” assays for bkv-miR-B1-5p (0.990 and 0.992, respectively). The no-template negative control did not reveal any amplification signal for the whole assay. For both types of assays, bkv-miR-B1-3p RT-PCR was negative with bkv-miR-B1-5p template and bkv-miR-B1-5p RT-PCR was negative with the bkv-miR-B1-3p template (data not shown). For each miRNA concentration, the Ct obtained using “advanced” RT-PCR was unexpectedly higher than those measured with the “classic” assay.

### 3.2 Qualitative performance of RT-qPCR assays in patients

A study previously reported that BKPyV miRNA quantification in urine could be relevant for viral monitoring of KTRs with presumptive BKVN because bkv-miR-B1-3p and bkv-miR-B1-5p indicate earlier than BKPyV DNA the resolution of BKVN (23, 34). Bkv-miR-B1-3p and bkv-miR-B1-5p were then quantified in the urine of KTRs using “advanced” and “classic” TaqMan RT-PCR assays. All 44 urine samples tested for BKPyV miRNA were positive for BKPyV DNA. First, the RT-PCR results were analyzed qualitatively by considering the results as positive or negative, independently of the Ct value and the corresponding concentration. As reported in Table 1, bkv-miR-B1-3p was found in 35 urine samples (79.54%) with the “classic” assay, whereas the “advanced” assay revealed only 15 (34.09%) positive urine samples for this miRNA (*p* < 0.001). Similar results were obtained with bkv-miR-B1-5p testing. In fact, bkv-miR-B1-5p was detectable in 37 (84.09%) and 7 (15.91%) urine samples after “classic” and “advanced” RT-PCR assay, respectively, which revealed a significant difference between proportions (*p* < 0.001). Overall, bkv-miR-B1-5p was less frequently detectable than bkv-miR-B1-3p with the “advanced” assay (*p* = 0.016). Though the proportions were equivalent to the “classic” assay (*p* = 0.78).

**Table 1:** Contingency of bkv-miR-B1-3p and bkv-miR-B1-5p detection in the urine of KTRs with BKPyV DNA positivity.

Urine samples BKPyV DNA + *n* = 44	bkv-miR-B1-3p + *n* (%)	bkv-miR-B1-5p + *n* (%)	Value of *p*
*TaqMan MicroRNA Assays*®	35 (79.54%)	37 (84.09%)	*p* = 0.78
*TaqMan Advanced MicroRNA Assays*®	15 (34.09%)	7 (15.91%)	***p* = 0.016**
**Value of *p***	***p* < 0.001**	***p* < 0.001**	

For each miRNA, RT-PCR was performed with *TaqMan MicroRNA Assays*® and *TaqMan Advanced MicroRNA Assays*®. The *p*-value was obtained from Fisher’s exact test. Bold values represent significant *p*-values.

**Figure 2:**
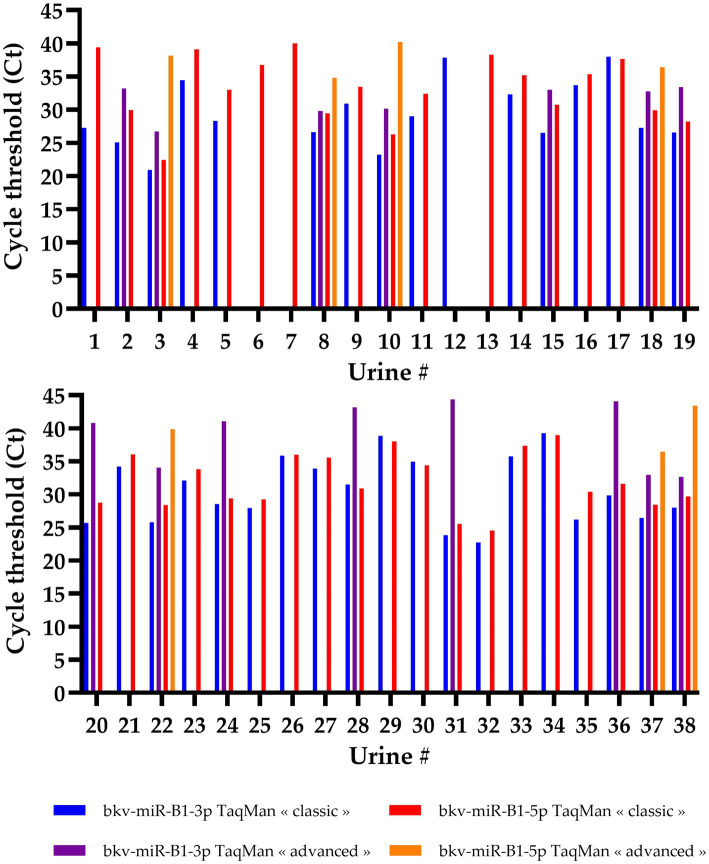
Cycle threshold (Ct) values of bkv-miR-B1-3p and bkv-miR-B1-5p RT-PCR assays. For each miRNA, RT-PCR was performed *with TaqMan MicroRNA Assays*® (called “classic”) and *TaqMan Advanced MicroRNA Assays*® (called “advanced”). Among the 44 tested urine samples, 6 were negative for both miRNAs and both assays and are not represented in this figure.

Individual analysis of the results for each sample was in line with the general trend (Figure 2). A total of 34 samples were positive for both bkv-miR-B1-3p and bkv-miR-B1-5p with “classic” RT-PCR. “Advanced” RT-PCR highlighted only seven samples positive for both miRNAs. Isolated detection of bkv-miR-B1-3p and bkv-miR-B1-5p was found in only one and three urine samples, respectively. This observation was exclusively performed with the “classic” RT-PCR assay, and the Ct values were > 35, suggesting very low quantities in the sample or an unspecific reaction. A detection of bkv-miR-B1-3p and/or bkv-miR-B1-5p with the “advanced” assay was necessarily associated with a detection by the “classic” assay. On the other hand, most “classic” detections of the miRNAs were not found with the “advanced” assay. Furthermore, when a miRNA was detected by both “classic” and “advanced” assays, Ct values obtained with the “advanced” RT-PCR were always higher than with the “classic” RT-PCR. These results unexpectedly suggest that *TaqMan Advanced MicroRNA Assays* present a lower sensitivity than *TaqMan MicroRNA Assays* to detect and quantify bkv-miR-B1-3p and bkv-miR-B1-5p in the urine of KTRs, despite BKPyV DNA positivity.

**Figure 3:**
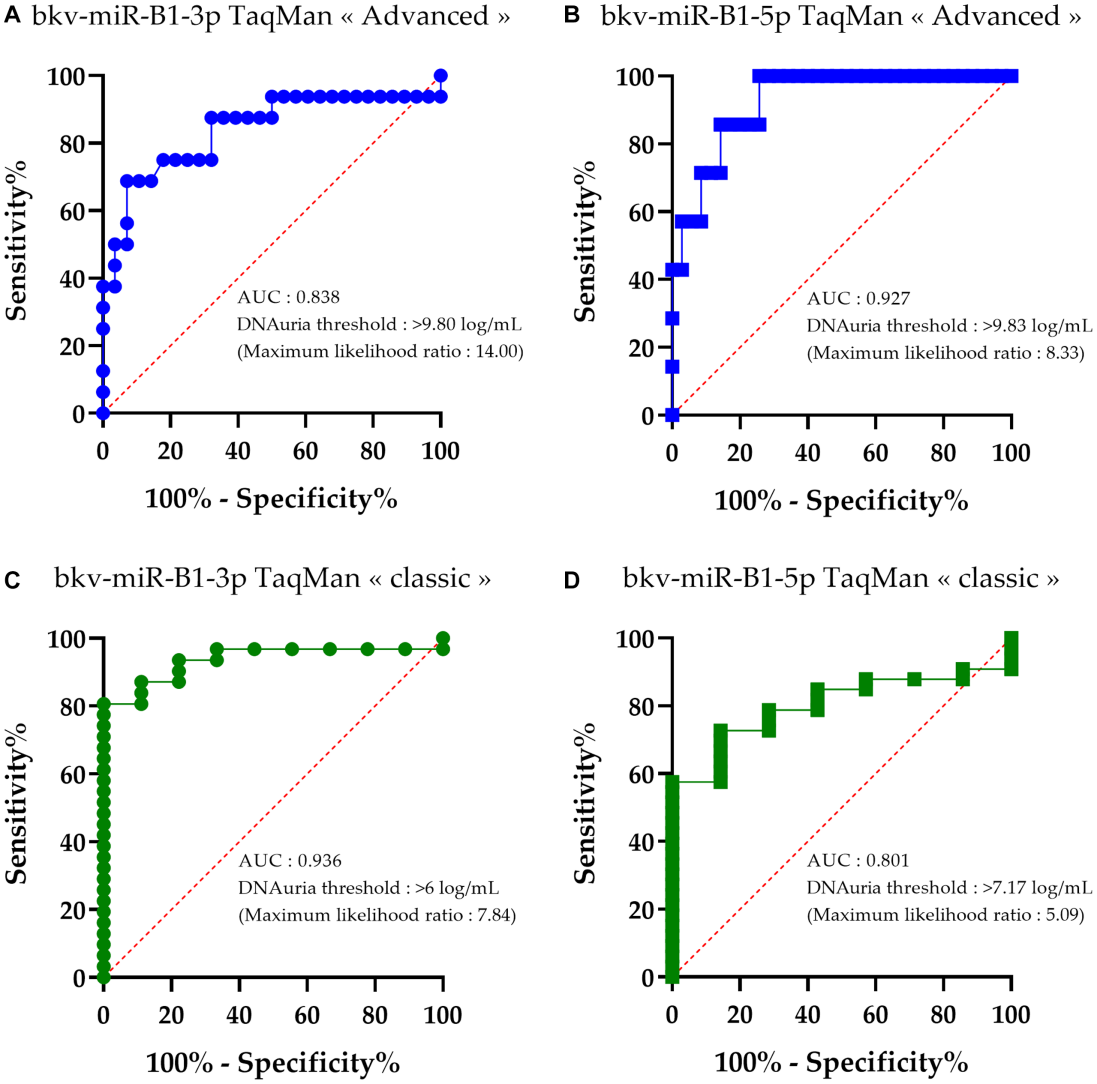
Receiver operating characteristic (ROC) curves of bkv-miR-B1-3p and bkv-miR-B1-5p detection with RT-PCR assays relative to BKPyV DNAuria levels. For each miRNA, RT-PCR was performed with *TaqMan MicroRNA Assays*® (called “classic”) and *TaqMan Advanced MicroRNA Assays*® (called “advanced”). Curves **(A)** and **(B)** correspond to performance of bkv-miR-B1-3p and bkv-miR-B1-5p detection, respectively, with *TaqMan Advanced MicroRNA Assays*®. Curves **(C)** and **(D)** correspond to performance of bkv-miR-B1-3p and bkv-miR-B1-5p detection, respectively, with *TaqMan MicroRNA Assays*®. AUC, area under the curve. Each point of the curves corresponds to the calculated “100%-specificity” and sensitivity to categorize the miRNA detection (positive or negative) based on an observed BKPyV DNAuria level.

In order to compare the deviation of diagnostic performance between “classic” and “advanced” RT-PCR assays, BKPyV DNAuria levels were sorted depending on the detection of bkv-miR-B1-3p or bkv-miR-B1-5p with the different assays. Then, receiver operating characteristic (ROC) curves were constructed to identify the DNAuria level that permitted the patients to distinguish whether they were positive or negative for each miRNA. As shown in Figure 3, areas under the curve (AUC) were significant for all tested methods and miRNAs. For each assay, sensitivity and specificity analyses lead to the determination of a BKPyV DNAuria threshold that corresponds to the best combination of sensitivity and specificity performances. This threshold was thus determined according to the maximum likelihood ratio.

The DNAuria thresholds to discriminate at best the samples positive for bkv-miR-B1-3p and bkv-miR-B1-5p with an “advanced” assay were high. In fact, the best DNAuria threshold to be concordant between the DNA load and miRNA detection in urine was 9.80 log copies/mL and 9.83 log copies/mL for bkv-miR-B1-3p and bk5p, respectively (Figures 3A,B). This means that the “advanced” RT-PCR assay confidently revealed BKPyV miRNA only when DNA is highly found in urine at the advanced stage of the viral disease.

The DNAuria thresholds to discriminate at best the samples positive for bkv-miR-B1-3p and bkv-miR-B1-5p with a “classic” assay were 6.00 log copies/mL and 7.17 log copies/mL, respectively (Figures 3C,D). These thresholds are concordant with the DNAuria level threshold defined to make the early diagnosis of possible BKVN, i.e., 7 log copies/mL.

### 3.3 Quantitative performance of RT-qPCR assays in patients

**Figure 4:**
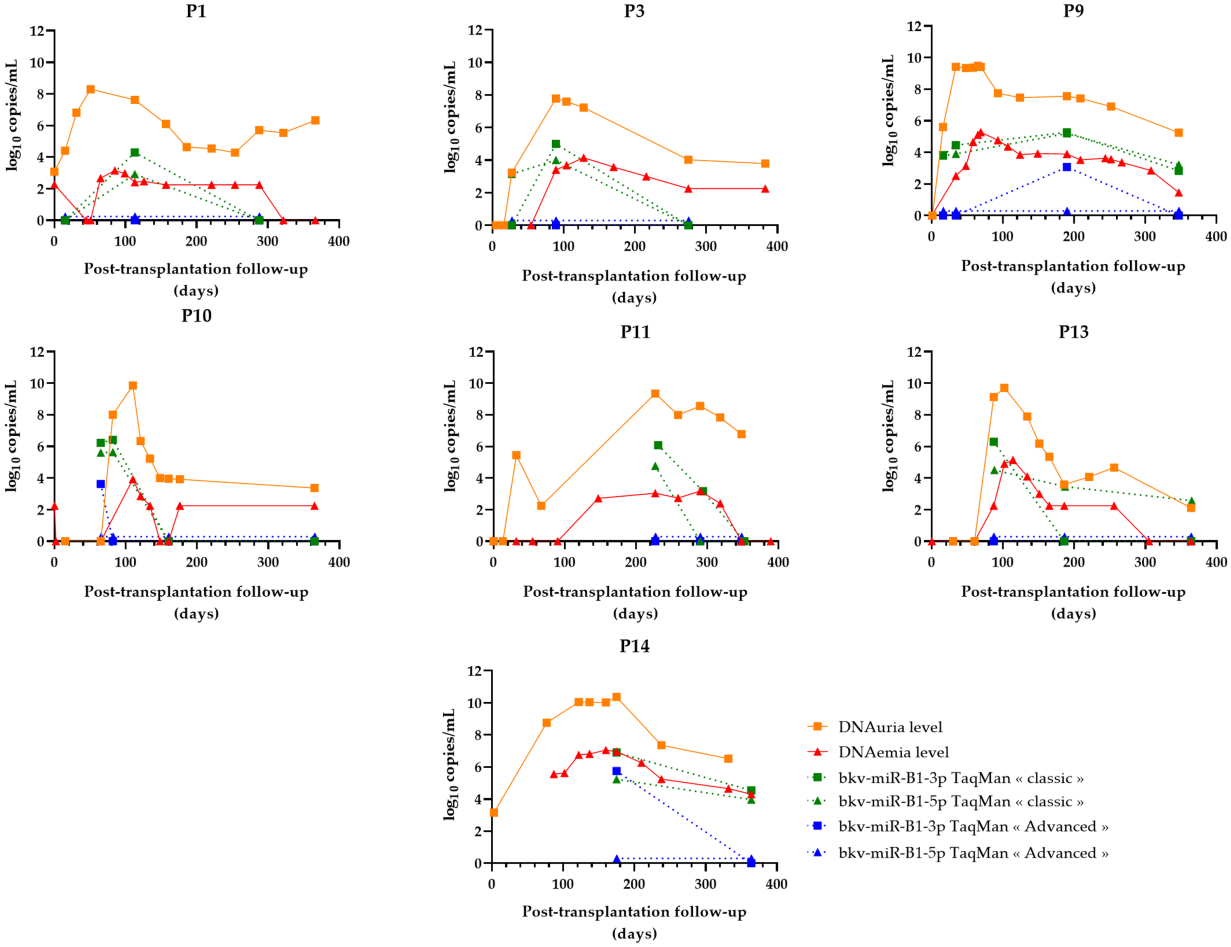
Changes over time of BKPyV markers (DNAuria, DNAemia, urine bkv-miR-B1-3p, and urine bkv-miR-B1-5p levels) among seven patients who resolved BKPyV replication during the year after kidney transplantation. DNAuria (orange squares) and DNAemia (red triangles) levels were collected from medical records. Bkv-miR-B1-3p (squares) and bkv-miR-B1-5p (triangles) quantification were performed applying *TaqMan MicroRNA Assays*® (called “classic”; represented in green) and *TaqMan Advanced MicroRNA Assays*® (called “advanced”; represented in blue).

**Figure 5:**
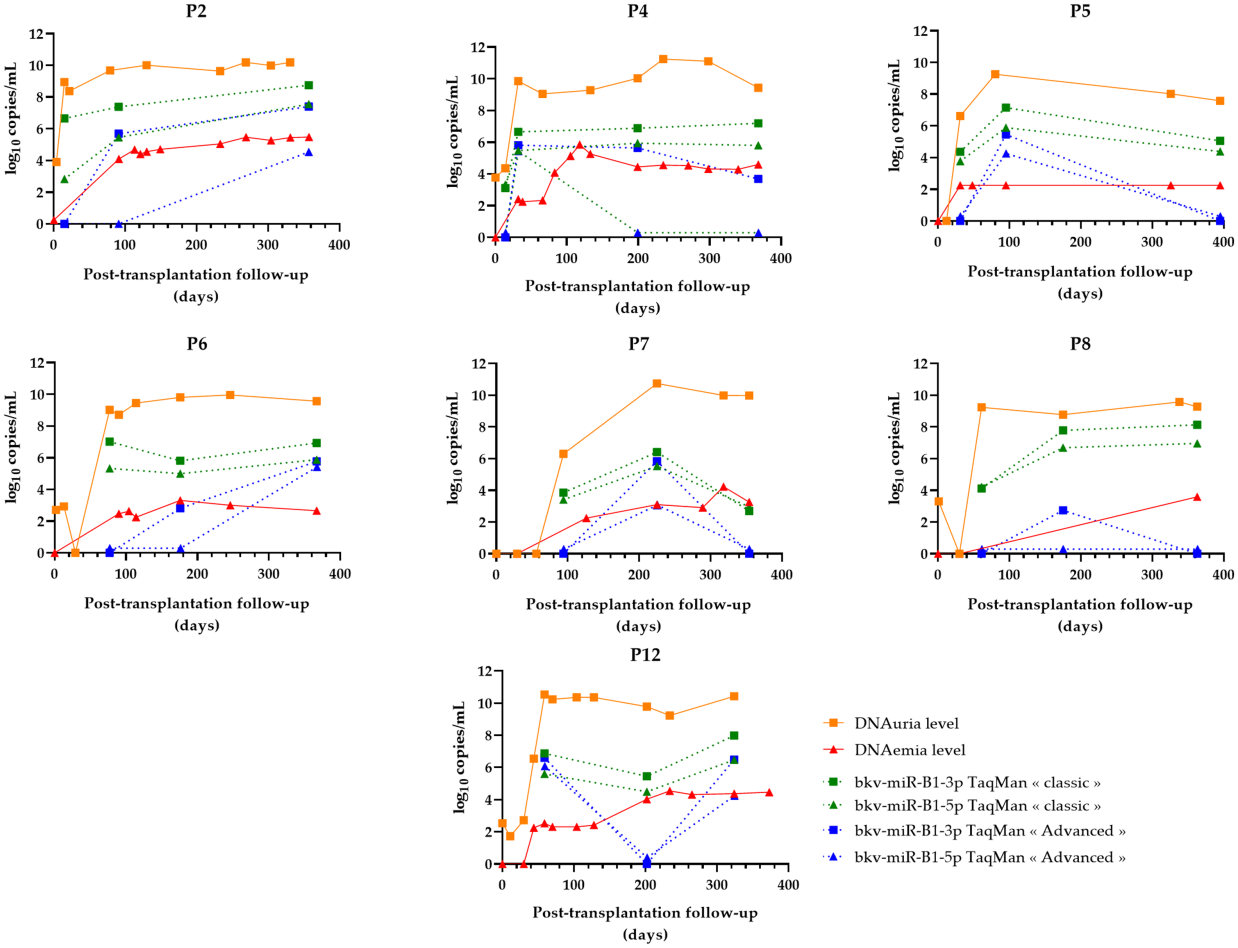
Changes over time of BKPyV markers (DNAuria, DNAemia, urine bkv-miR-B1-3p, and urine bkv-miR-B1-5p levels) among seven patients who developed sustained BKPyV replication during the year after kidney transplantation. DNAuria (orange squares) and DNAemia (red triangles) levels were collected from medical records. Bkv-miR-B1-3p (squares) and bkv-miR-B1-5p (triangles) quantification were performed applying *TaqMan MicroRNA Assays*® (called “classic”; represented in green) and *TaqMan Advanced MicroRNA Assays*® (called “advanced”; represented in blue).

As the 44 urine samples were obtained from the longitudinal follow-up of 14 KTRs with probable BKVN, quantitative analysis of RT-PCR results permitted a comparison of the individual progress of the BKPyV miRNA level according to the applied technique. BKPyV DNAuria and DNAemia levels, prospectively measured during follow-up, were collected from the medical records. For all 14 patients, these data were then put in relation to the quantification of urine bkv-miR-B1-3p and bkv-miR-B1-5p levels obtained using “classic” and “advanced” RT-qPCR assays. In a previous study, we observed a different progression of BKPyV miRNA levels between patients with resolved BKVN compared to those with sustained BKVN, as defined in the Methods section. Interestingly, we reported an early decrease of miRNA levels in KTRs with resolved BKVN, compared to BKPyV DNA levels that diminished more slowly (23). Thus, in the present study, the time courses of BKPyV biomarkers were grouped for patients with resolved BKVN (Figure 4) or sustained BKVN (Figure 5).

One of the challenges when measuring the miRNA load in the urine of KTRs with BKPyV replication is having a more specific marker of replication than viral DNA (23). The aim is to use these viral molecules as an objective and precise tool to adjust an immunosuppressive regimen or manage a specific antiviral therapy when it becomes available. Since no validated method exists for BKPyV miRNA quantification, nephrologists and virologists need experimental data obtained with different methods and populations to consider applying in real-life practice. By retrospectively analyzing the evolution of viral markers for each patient according to the type of replication (sustained or resolutive), the relevance of “classic” and “advanced” RT-qPCR assays could be compared.

In the case of resolved infections (Figure 4), a decrease in miRNAs was observed in the same way as DNA in 100% of cases with the “classic” technique. Among these patients, three patients showed an early negativization of miRNAs compared to DNA (patients P1, P3, and P10). A negativation of bkv-miR-B1-3p was also observed for patient P13, with persistently low levels of bkv-miR-B1-5p despite undetectable DNA at the end of the follow-up. For the “advanced” RT-qPCR, all seven resolutive BKVN cases were marked by a low or undetectable level of BKPyV miRNA at the moment or following the diagnosis of probable BKVN. Thus, by using this technique, early negativation of urine BKPyV miRNA compared to DNAuria and DNAemia is less obvious, but prolonged undetectable miRNAs could be considered good prognostic markers.

For sustained BKVN cases (Figure 5), the “classic” RT-qPCR technique showed stable or increasing urine miRNA levels in four of seven (57%) patients (P2, P4, P6, and P8). However, the accuracy of the “advanced” RT-qPCR was lower to attest to the continuation of BKPyV replication. In fact, miRNA levels raised or remained stable for only two of seven (29%) KTRs.

The prediction of the progression of the infection (sustained or resolved) based on the miRNA level course is summarized in Table 2. For the two evaluated assays, symbols were assigned according to the clinical impact of the marker dynamics to follow the infection. Two situations were identified: 1/ unfavorable situations for the marker (marked by “◊” symbol in Table 2) which entail a risk of wrongly diagnosing a controlled infection, which would lead to an erroneous adjustment of anti-rejection treatments or, on the contrary, unnecessarily maintaining low doses of immunosuppressive drugs in the case of a resolved infection; and 2/ situations when the changes in miRNA levels are concordant with the infection outcome (highlighted with “†” symbol in Table 2). According to this contingency, both “classic” and “advanced” RT-qPCR methods lead to 100% positive predictive values. Nevertheless, the “classic” assay outperformed the “advanced” as negative predictive values were 70 and 58%, respectively. The overall qualitative and quantitative assessments of bkv-miR-B1-3p and bkv-miR-B1-5p RT-qPCR on clinical samples are therefore in agreement with the preliminary synthetic miRNA assays: the *TaqMan MicroRNA Assays*® showed better sensitivity than the *TaqMan Advanced MicroRNA Assays*®.

**Table 2:** Contingency BKPyV miRNA level progression in the urine of KTRs with sustained or resolved BKVN.

	Sustained BKVN (*n* = 7)		Resolved BKVN (*n* = 7)		
BKPyV miRNAs progression	Stable or increasing ◊	Decreasing or negativation †	Stable or increasing †	Decreasing or negativation ◊	Undetectable during the entire follow-up ◊
TaqMan MicroRNA assay ®	*n* = 4 P2, P4, P6, P8	*n* = 3 P5, P7, P12	*n* = 0	*n* = 7 P1, P3, P9, P10, P11, P13, P14	*n* = 0
TaqMan Advanced MicroRNA assay ®	*n* = 2 P2, P6	*n* = 5 P4, P5, P7, P8, P12	*n* = 0	*n* = 3 P9, P10, P14	*n* = 4 P1, P3, P11, P13

Analyses were performed according to miRNA levels measured using RT-qPCR with *TaqMan MicroRNA Assays®* and *TaqMan Advanced MicroRNA Assays*®. The table summarizes the number and identification of cases described in Figures 4, 5.

## 4 Discussion

In the present study, *TaqMan MicroRNA Assays®* outperformed *TaqMan Advanced MicroRNA Assays®* to detect and quantify bkv-miR-B1-3p and bkv-miR-B1-5p in laboratory and clinical samples. *TaqMan microRNA Assays* use a simple two-step protocol that requires a novel target-specific stem-loop primer for reverse transcription (RT) and cDNA synthesis to produce a template that is used subsequently for real-time PCR with the corresponding TaqMan probe and primer set. With this assay, a two-step RT-PCR is required for each target miRNA, which ensures specific amplification despite substantial costs and time when several miRNAs are sought in a single eluate. *TaqMan Advanced miRNA Assays* use a universal RT step for a streamlined workflow, which amplify the whole miRNAs contained in the eluate in a single reaction. Then, a universal miR-Amp step enables, theoretically, highly sensitive detection using the subsequent specific real-time PCR performed for each targeted miRNA and more limited costs when the detection of multiple miRNAs is needed (42, 43). However, the manufacturer described the equivalent or better performance of *TaqMan Advanced MicroRNA Assays*® to detect several human miRNAs (has-miR-21, has-miR-103, has-miR-16, has-miR-125b, and has-let-7f) purified from cell cultures (43). To our knowledge, these results have not been published through the peer-reviewing process. Published literature about the comparison between “classic” and “advanced” TaqMan RT-qPCR is scarce. In an article by Krepelkova et al., the authors compared these techniques for miR-142-5p detection in blood samples (42). They observed a lower sensitivity of *TaqMan Advanced MicroRNA Assays®* and described on calibration curves a 5.6-average Ct shift with the *TaqMan MicroRNA Assays®*. Based on our results, the average Ct shift between “classic” and “advanced” RT-PCR obtained for bkv-miR-B1-3p and bkv-miR-B1-5p calibration curves was 2.9 and 5.7, respectively. As the “advanced” assay detected less viral miRNA in urine samples than the “classic” assay, this Ct shift calculation was not relevant to the results on clinical samples. Another article by Sequeira et al. showed lower efficiency of the “advanced” TaqMan assay to amplify has-miR-371a-3p (44). This miRNA is known to be overexpressed in the case of testicular germ cell tumors. In this study, the frequency of has-miR-371a-3p detection in tumoral tissue was inferior with the “advanced” assay in comparison to the “classic” assay. In fact, this miRNA was detected using “advanced” RT-PCR only for advanced tumoral stages, whereas “classic” RT-PCR amplified it even for early stages of the disease. In the same way, for BKPyV miRNA RT-qPCR, we observed that miRNAs were detected using the “advanced” assay only when the DNAuria level was high, which is usually associated with severe infection. As Sequeira et al. did in their article, we warn colleagues working on BKPyV miRNA that miRNA RT-qPCR assays must be compared and evaluated according to the specific purpose of the studies. Moreover, due to the limitation on the volume of clinical samples, it was not possible to check the reproducibility of the measurements. This uncertainty could affect the interpretation of the results in prospective studies or routine applications and should be evaluated. To ensure high reproducibility, the development of international bkv-miR-B1-3p and bkv-miR-B1-5p standards will be required, as was done for BKPyV DNA PCR (45). Recently, Suthanthiran et al. developed a method for standard absolute quantification of BKPyV-VP1 mRNA in the urine of KTRs (46), another unconventional marker of productive viral infection. This method was used to assess the non-invasive diagnosis of BKVN with high performance (47). Although mRNA is known to be less stable in urine than miRNA (37, 48), mRNA quantification often requires pre-analytical arrangements. A direct comparison between BKPyV-VP1 mRNA and BKPyV miRNA quantification assays would be interesting, notably with longitudinal follow-up of KTRs to observe the changes in nucleic acid loads in sustained and resolved BKVN.

Differences in performance between the two assays for BKPyV miRNA quantification influenced the analysis of BKVN patients’ biological follow-up. In fact, the “advanced” RT-qPCR assay was less relevant than the “classic” assay to monitor BKPyV replication in KTRs and for the early identification of resolutive and good-prognosis infections. However, the population analyzed in the present study was small, with groups of only seven patients. This is related to the short period of inclusion (1 year) and frequency of BKPyV replication in KTRs. Moreover, we aimed to describe the time course of miRNA levels, which required multiple leftovers of urine for each patient, so we had to exclude some patients because of insufficient samples in this retrospective study. Though, the large number of urine samples (*n* = 44) is sufficient to assess the default in terms of sensitivity of *TaqMan Advanced MicroRNA Assays®.* Nevertheless, the limits of this technique, according to the calculated negative predictive values, must be nuanced because of the small number of BKVN cases included in this study. Because the contingencies were done in relation to the time course of miRNA levels during the follow-up, the results could be influenced by the time points available and analyzed retrospectively. In fact, the number of measurements and the delay between them were heterogeneous. A higher number of measurements and more precise curves of miRNA progression are essential for clinical relevance evaluation. A prospective study on a larger cohort is required to assess the usefulness of urine BKPyV miRNA quantification as a relevant marker of viral replication in KTRs.

The sensitivity of the quantification of BKPyV miRNA in the urine of KTRs was one of the main points discussed in a previous study (23). Unfortunately, the “advanced” assay did not show better results than the “classic” one, which was used in the above-mentioned article. Indeed, the sensitivity of the “classic” assay was unexpectedly better, in spite of the pre-amplification step of the “advanced” assay. The higher frequency of detection of the viral miRNA with the “classic” assay may be due to a lack of specificity, as this technique is based on specific RT primers, whereas the “advanced” assay requires universal RT primers. Moreover, we found few discrepancies between the bkv-miR-B1-3p and bkv-miR-B1-5p detection, and it was also linked to the isolated bkv-miR-B1-5p detection, which is supposed to be specific to BKPyV. The only isolated bkv-miR-B1-3p detection was probably not a cross-specific reaction with jcv-miR-J1-3p because the absence of JCPyV DNA in selected urine samples was verified before inclusion. As usual, since no method is commonly considered the gold standard, specificity, sensitivity, and negative and positive predictive values of assays depend on the study design and must be carefully interpreted.

Hence, overpassing the limitations of miRNA RT-qPCR is still challenging, and the potential of miRNA as a minimally invasive biomarker has not yet been converted into clinical practice. Conventional RT-qPCR used to be the standard method, but RNA extraction is labor/resource-intensive, and amplification could be biased by imperfect specificity, limited sensitivity, or poor reproducibility. Moreover, the detection of miRNA using RT-qPCR may be discordant with the results obtained with other technologies, questioning the rightfulness of RT-qPCR as a standard method to compare with (49). Many technologies in development depend on amplification and/or extraction-free methods. For example, Cai et al. developed an electro-optical sensing platform that allows the detection of miRNAs directly in small volumes of serum samples without amplification (50). The sensitivity was in the femtomolar range, and the method was multiplexable and single-base mismatch selective. Other authors described the amplification-free electrochemical detection of urinary miRNA for diabetic nephropathy that might be analyzed with the naked eye, spectrophotometry, or electrochemistry (51). The limits of detection were 1 pM, 6 fM, and 0.65 fM, respectively, highlighting the possibility of point-of-care application without enhancing the usual sensitivity of RT-qPCR. Femtomolar sensitivity can also be reached with DNA cascade reactors that functionalize photonic crystal arrays within a limited time (15 min) in a point-of-care testing manner (52). Rolling circle amplification, loop-mediated isothermal amplicfication, and strand-displacement amplification are nucleic acid amplification-based methods widely applied for highly sensitive miRNA detection (53). Basically, next-generation sequencing might be a reliable technology to detect multiple miRNAs, but the limits of detection and normalization of quantitative results are variable (49). However, the clinical application of miRNA detection as a disease biomarker seems closer when digital droplet PCR (ddPCR) is used (54). The sensitivity is equivalent to or better than conventional PCR and allows absolute quantitation (44, 55). ddPCR therefore frees us from the need to standardize the results to internal or external process controls, even if the evaluation of the quality of the nucleic acid extraction remains essential (56, 57).

## Data availability statement

The raw data supporting the conclusions of this article will be made available by the authors, without undue reservation.

## Ethics statement

The studies involving humans were approved by Ethics Committee of the Amiens University Medical Center (protocol code PI2022_843_0078, 2022 May 12th). The studies were conducted in accordance with the local legislation and institutional requirements. The participants provided their written informed consent to participate in this study.

## Author contributions

KZ: Data curation, Formal analysis, Investigation, Writing – original draft. VD: Methodology, Supervision, Writing – review & editing. AA: Formal analysis, Visualization, Writing – review & editing. FH: Writing – review & editing. CF: Methodology, Project administration, Resources, Writing – review & editing. SC: Writing – review & editing. EB: Conceptualization, Supervision, Validation, Writing – review & editing. BD: Conceptualization, Methodology, Supervision, Writing – review & editing.
